# *Mycobacterium tuberculosis* Infection of Domesticated Asian Elephants, Thailand

**DOI:** 10.3201/eid1612.100862

**Published:** 2010-12

**Authors:** Taweepoke Angkawanish, Worawidh Wajjwalku, Anucha Sirimalaisuwan, Sittidet, Mahasawangkul, Thattawan Kaewsakhorn, Kittikorn Boonsri, Victor P.M.G. Rutten

**Affiliations:** Author affiliations: National Elephant Institute, Forest Industry Organization, Lampang, Thailand (T. Angkawanish, S. Mahasawangkul);; Kasetsart University, Nakhonpathom, Thailand (W. Wajjwalku);; Chiang Mai University, Chiang Mai, Thailand (A. Sirimalaisuwan, T. Kaewsakhorn, K. Boonsri);; Utrecht University, Utrecht, the Netherlands (T. Angkawanish, V.P.M.G. Rutten);; University of Pretoria, Onderstepoort, South Africa (V.P.M.G. Rutten)

**Keywords:** Asian elephant, Thailand, *Mycobacterium tuberculosis*, bacteria, dispatch

## Abstract

Four Asian elephants were confirmed to be infected with *Mycobacterium tuberculosis* by bacterial culture, other diagnostic procedures, and sequencing of 16S–23S rDNA internal transcribed spacer region, 16S rRNA, and gyrase B gene sequences. Genotyping showed that the infectious agents originated from 4 sources in Thailand**.** To identify infections, a combination of diagnostic assays is essential.

During the past 2 decades, infections of captive African and Asian elephants with *Mycobacterium bovis* and *M. tuberculosis* have been diagnosed worldwide (*1**–**4*). Transmission of these infections to other mammals and veterinary personnel has also been observed (*5*). To date, *M. tuberculosis* infection has not been reported in elephants in Thailand. Four elephants referred to the National Elephant Institute (NEI) Hospital during 2005–2008, three of which showed signs of weakness and chronic weight loss, and 1 showed serous nasal discharge. Tuberculosis was confirmed by using conventional and molecular diagnostic assays.

## The Study

The ElephantTB Stat-Pak (Chembio Diagnostic Systems, Inc, Medford, NY, USA), which detects antibodies specific to M. tuberculosis in elephants, was performed. Trunk wash sampling of elephants 1, 2, and 4, according to the Guidelines for the Control of Tuberculosis in Elephants, 2008 (6), was followed by culture for bacteria (Technical Appendix Table 1). Necropsy of elephants 1, 3, and 4 was performed at 21 months, 7 days, and 33 months after admission, respectively, and lesion tissues were collected for bacterial culture, Ziehl-Neelsen (ZN) staining, and histopathologic examination (Technical Appendix Table 2 ). 

A serum sample from elephant 1 was negative for *M. tuberculosis* at admission, but a sample obtained 10 months later was positive. Bacteria could not be grown from trunk wash samples. Necropsy showed that elephant 1 had tuberculous lesions in the respiratory tract, mediastinal lymph nodes, liver, kidney, and spleen. Histopathologic examination showed caseous necrosis; infiltration of lymphocytes; and accumulation of macrophages and giant cells in lung tissue, lymph nodes, and liver. ZN staining identified acid-fast bacilli. Mycobacteria were cultured from lesion tissue.

A serum specimen from elephant 2 was negative for mycobacteria at admission, but a second sample was positive 23 months later. Bacteria that were positive by ZN staining were cultured from a trunk wash sample. This elephant is still alive and being kept in a restricted area.

Serum samples from elephant 3 were negative at days 1 and 7 after admission, and the elephant died a few hours after the second sample was tested. A stored serum sample from elephant 3, obtained 4 months earlier was also negative. The animal was severely ill and in lateral recumbency. Necropsy showed tuberculous lesions in the lungs, upper trachea, and mediastinal lymph nodes. Histopathologic examination showed caseous necrosis and accumulation of macrophages and giant cells in the lung and lymph nodes. ZN staining showed acid-fast bacilli. Mycobacteria were cultured from lesion tissues.

A serum specimen from elephant 4 was positive at admission. Initially, *M. avium* bacteria were grown from cultures of trunk wash samples. At necropsy, tuberculous lesions were found in the respiratory organs and mediastinal lymph nodes. Histopathologic examination showed accumulation of macrophages and edema in the lung tissues. ZN staining did not show acid-fast bacilli. However, mycobacteria were cultured from lesion tissues.

Bacteria cultured from trunk wash and tissue samples were further identified by PCR reactions by using 16S rRNA, 16S–23S-rDNA internal transcribed spacers (ITS) (*7**,**8*), and gyrase B (*gyrB*) primers (Table 1). The subsequent sequencing was conducted by using an ABI 3070 system (Applied Biosystems, Foster City, CA, USA). Unambiguous sequences were compared with data available in GenBank (www.ncbi.nih.gov/BLAST) and analyzed by using ClustalW version 1.4 (www.ebi.ac.uk/Tools/clustalw/). The 16S rRNA and ITS sequencing confirmed that bacteria from lesion tissues of elephants 1, 3, and 4 and from a trunk wash sample of elephant 2 belong to the *M. tuberculosis* complex. The *gyrB* sequences of isolates from elephants 2, 3, and 4 were identical to those of *M. tuberculosis* strain American Type Culture Collection (ATCC) 27294 and others (Table 2); the *gyrB* sequence of the isolate from elephant 1 differed at position 482, which is similar to the *M. tuberculosis* strain KPM KY679, the ancient TbD_1_-positive strain (*9**–**11*). The mycobacterial interspersed, repetitive-unit variable number tandem repeat typing of the exact tandem repeat-A (ETR-A) locus was performed according to protocols of Fleche et al. (*12*). The sequence of the ETR-A locus showed that different types of *M. tuberculosis* were present in elephants 2, 3, and 4 because the sequence had 3, 2, and 4 repeats of the typical 75-bp sequence, respectively.

**Table 1:** Primers used to identify bacteria cultured from trunk wash and tissue samples from domesticated Asian elephants, Thailand, 2003–2008*

Primer	Forward	Reverse
16s rRNA	5′-AgA gTT TgA TCC Tgg CTC Ag-3′	5′-ACg gCT ACC TTg TTA CgA CTT-3′
ITS	5′-TTg TAC ACA CCg CCg gTC a-3′	5′-TCT CgA TgC CAA ggC ATC CAC C-3′
*gyrB*	5′-TCG GAC GCG TAT GCG ATA TC-3′	5′-ACA TAC AGT TCG GAC TTG CG-3′

*ITS modified from (*7**,**8*); *gyrB* modified from (*9*). ITS, internal transcribed spacer; *gyrB*, gyrase B.

**Table 2:** *gyrB* gene sequence comparisons of 4 *Mycobacterium tuberculosis* isolates from domesticated Asian elephants, Thailand, 2003–2008*

Organism	Gene position						
	41	122	482	677	776	816	824
*M. tuberculosis* ATCC	C	G	G	T	C	G	C
*M. tuberculosis* TbD1	C	G	C	T	C	G	C
*M. africanum*	C	G	G	T	C	T	C
*M. canetti*	C	G	G	T	C	T	T
*M. microti*	T	G	G	T	C	T	T
*M. bovis*	C	A	G	T	T	T	T
*M. caprae*	C	A	G	G	T	T	T
Elephant 1 isolate	C	G	C	T	C	G	C
Elephant 2 isolate	C	G	G	T	C	G	C
Elephant 3 isolate	C	G	G	T	C	G	C
Elephant 4 isolate	C	G	G	T	C	G	C

*Nucleotide variability at relevant positions of the *gyr B* gene in the genome of mycobacteria isolated from the 4 infected elephants as compared with those in established *M. tuberculosis* ATCC 27294 (GenBank accession no. GQ247736.1), *M. tuberculosis* KPM KY679 (accession no. AB014215.1), *M. africanum* (accession no. AB014192.1), *M. canetti* (accession no. AJ749915.1), *M. microti* (accession no. AB014205.1), *M. bovis* (accession no. AB018554.1), and *M. caprae (*accession no. AJ276122.1). Modified from Gutierrez et al***.*** (*11*). Shading corresponds to sequence stretch in the strains that are identical to the sequences in *M. tuberculosis*: yellow, performed ATCC strain; blue, position 482 performed TbD1 strain; and tan, performed other *M. tuberculosis* complex strain. *gyrB*, gyrase B; ATCC, American Type Culture Collection.

## Conclusions

We report *M. tuberculosis* infection in elephants in Thailand. Clinical signs shown by these 4 elephants varied considerably. Elephant 2 showed nasal discharge only; in contrast, elephant 3, showed severe clinical signs and lateral recumbency. Elephant 3 had no antibodies, which may indicate an anergic status of the mycobacteria-specific immune response (*13*). Histopathologic examination showed that this elephant was severely affected by the infection. Elephant 2 is still alive; cultures of trunk wash samples contain mycobacteria. The elephant was seropositive for *M. tuberculosis* antigens as defined by the *StatPak* assay. The other 2 elephants (1 and 4) showed anorexia, chronic weight loss, and comparable lesions at necropsy, but diagnostic assays showed variable results. Trunk wash culture, considered to be the standard for confirmation of *M. tuberculosis* complex infection in elephants, has its limitations, as described elsewhere (*14*). This study, which included 3 elephants positive for mycobacteria in tissue culture at necropsy, showed that bacterial cultures of only 2 of 60 trunk wash samples were positive for mycobacteria. The study indicates that serologic tests or other diagnostic procedures could not unequivocally identify infected animals, perhaps because of differences in specific immune responsiveness among species and length of time after infection (*13*). However, the combination of the different diagnostic observations after infection holds promise for improving the likelihood of confirmed *M. tuberculosis* infection.

Sequence analysis of 16S and ITS indicated *M. tuberculosis* complex bacteria in each elephant. Nucleotide sequence polymorphism in the *gyrB* gene of the mycobacteria isolates (*9**–**11*) confirmed the identity of *M. tuberculosis* for all 4 elephants. *M. tuberculosis* may be classified into ancestral and modern strains based on *M. tuberculosis*–specific deletion (TbD1) (*15*). *M. tuberculosis* isolated from elephant 1 had a *gyrB* gene sequence identical to strains of the ancient TbD_1_-positive strain (Table 2). The other 3 elephants were infected with strains identical to *M. tuberculosis* ATCC 27294, the modern type, potentially related to major epidemics like the Beijing, Haarlem, and African *M. tuberculosis* clusters (*15*).

On the basis of these molecular studies, we believe that *M. tuberculosis* was probably transmitted to these 4 elephants from humans. In addition, mycobacterial interspersed, repetitive-unit variable-number tandem-repeat typing of the ETR-A gene *M. tuberculosis* strains in elephants 2, 3, and 4 showed different numbers of the typical 75-bp repeat. Therefore, we conclude that the sources of infection were of different origins. Annual health checks of mahouts and veterinarians who were in contact with the infected animals for >4 years at the NEI did not identify any persons with positive results by chest radiograph when tested as part of the tuberculosis control program in Thailand. To control *M. tuberculosis* complex transmission from humans and other species to wild animals, including elephants, or from wild animals to humans, assays that enable early diagnosis of infection are necessary. Because no assay unequivocally defines the infectious status, a combination of diagnostic approaches is essential.

Further investigation of tuberculosis transmission and surveillance and monitoring of this disease in Thailand will enhance the understanding of its epidemiology. Increased epidemiologic knowledge is essential to control and prevent tuberculosis in elephants.

## Supplementary Material

Technical Appendix Comparison of results of bacterial culture from trunk washes at various times during hospitalization and from tissue samples obtained at necropsy as well as of serology in course of time and Clinical signs at and during hospitalization and gross and microscopic lesions as well as ZN-positive bacilli observed at and after necropsy.

## References

[1] Mikota SK, Peddie L, Peddie J, Isaza R, Dunker F, West G, Epidemiology and diagnosis of *Mycobacterium tuberculosis* in captive Asian elephants (*Elephas maximus*). J Zoo Wildl Med. 2001;32:1–16. 12790389 10.1638/1042-7260(2001)032[0001:EADOMT]2.0.CO;2

[2] Payeur JB, Jarnagin JL, Marquardt JG, Whipple DL. *Mycobacterium* isolation in captive elephants in USA. Ann N Y Acad Sci. 2002;969:256–8. 10.1111/j.1749-6632.2002.tb04388.x 12381601

[3] Michalak K, Austin C, Diesel S, Bacon JM, Zimmerman P, Maslow JN. *Mycobacterium tuberculosis* infection as a zoonotic disease: transmission between humans and elephants. Emerg Infect Dis. 1998;2:283–7. 10.3201/eid0402.980217 9621200 PMC2640151

[4] Lewerin SS, Olsson SL, Eld K, Röken B, Ghebremichael S, Koivula T, Outbreak of *Mycobacterium tuberculosis* infection among captive Asian elephants in a Swedish zoo. Vet Rec. 2005;156:171–5. 15736698 10.1136/vr.156.6.171

[5] Une Y, Mori T. *Tuberculosis* as a zoonosis from a veterinary perspective. Comp Immunol Microbiol Infect Dis. 2007;30:415–25. 10.1016/j.cimid.2007.05.002 17706284

[6] The National Tuberculosis Working Group for Zoo and Wildlife Species. Guidelines for the control of tuberculosis in elephants, 2008 [cited 2010 Oct 2]. http://www.aphis.usda.gov/ac/ElephTBGuidelines2008.html

[7] Weisburg WG, Barns SM, Pelletier DA, Lane DJ. 16S Ribosomal DNA amplification of phylogenetic study. J Bacteriol. 1991;173:697–703. 1987160 10.1128/jb.173.2.697-703.1991 PMC207061

[8] Jones SE, Shade AL, McMahon KD, Kent AD. Comparison of primer set for use in automated ribosomal intergenic space analysis of aquatic bacterial communities: an ecological perspective. Appl Environ Microbiol. 2007;73:659–62. 10.1128/AEM.02130-06 17122397 PMC1796982

[9] Kasai H, Ezaki T, Harayama S. Differentiation of phylogenetically related slowly growing *Mycobacteria* by their *gyrB* sequences. J Clin Microbiol. 2000;38:301–8. 10618105 10.1128/jcm.38.1.301-308.2000 PMC88713

[10] Gutierrez MC, Brisse S, Brosch R, Fabre M, Omais B, Marmiesse M, Ancient origin and gene mosaicism of the progenitor of *Mycobacterium tuberculosis.* PLoS Pathog. 2005;1:e5. 10.1371/journal.ppat.0010005 16201017 PMC1238740

[11] Niemann S, Harmsen D, Rusch-Gerdes S, Richter E. Differentiation of clinical *Mycobacterium tuberculosis* complex isolated by *gyrB* DNA sequence polymorphism Analysis. J Clin Microbiol. 2000;38:3231–4. 10970363 10.1128/jcm.38.9.3231-3234.2000 PMC87363

[12] Fleche PL, Fabre M, Denoueud F, Koeck JL, Vergnaud G. High resolution, on-line identification of strains from the *Mycobacterium tuberculosis* complex based on tandem repeat typing. BMC Microbiol. 2002;2:1471–2180. 2180–2-37. 10.1186/1471 PMC140014 12456266

[13] Van Rhijn I, Godfroid J, Michel A, Rutten VP. Bovine tuberculosis as a model for human tuberculosis: advantages over small animal models. Microbes Infect. 2008;10:711–5. 10.1016/j.micinf.2008.04.005 18538612

[14] Lyashchenko KP, Greenwald R, Esfandiari J, Olsen JH, Ball R, Dumonceaux G, Tuberculosis in elephants: antibody responses to defined antigens of *Mycobacterium tuberculosis*, potential for early diagnosis, and monitoring of treatment. Clin Vaccine Immunol. 2006;13:722–32 . 10.1128/CVI.00133-06 16829608 PMC1489565

[15] Brocsh R, Gordon SV, Marmiesse M, Brodin P, Buchrieser C, Eiglmeier K, A new evolutionary scenario for the *Mycobacterium tuberculosis* complex. Proc Natl Acad Sci U S A. 2002;6:3684–9. 10.1073/pnas.052548299 PMC122584 11891304

