# Vol. 16, No. 12

**DOI:** 10.3201/eid1704.C11704

**Published:** 2011-04

**Keywords:** errata, erratum, correction

An online Technical Appendix was omitted from the article *Mycobacterium tuberculosis* Infection of Domesticated Asian Elephants, Thailand (T. Angkawanish, et al.). The article has been corrected online (http://wwwnc.cdc.gov/eid/article/16/12/10-0862_article).

