# Supplementary material for: Mycobacterium tuberculosis Infection of Domesticated Asian Elephants, Thailand
Source: Emerg Infect Dis. 2010 Dec;16(12):1949–51. doi: 10.3201/eid1612.100862 (PMC3294569; doi:10.3201/eid1612.100862)
Supplement: Technical Appendix — Comparison of results of bacterial culture from trunk washes at various times during hospitalization and from tissue samples obtained at necropsy as well as of serology in course of time and Clinical signs at and during hospitalization and gross and microscopic lesions as well as ZN-positive bacilli observed at and after necropsy. [file 10-0862-Techapp_1p.pdf]

Article DOI: 10.3201/eid1612.100862

Mycobacterium tuberculosis Infection of Domesticated Asian Elephants, Thailand

Technical Appendix Table 1. Comparison of results of bacterial culture from trunk washes at various times during hospitalization and from tissue samples obtained at necropsy as well as of serology in course of time*

Elephant ID	Serology results (sampling times)		Bacteriological result (sampling times)	
	Negative	Positive	Negative (all trunk washes)	Positive
1	m 1	m 11<br>m 13<br>m 20<br>m 21	m 12<br>m 13<br>m 14<br>m 20<br>m 21	Necropsy at m 21: Lesional tissues (lung and mediastinal lymph nodes)
2	m 1	m 23<br>m 33<br>m 35<br>m 43<br>m 45<br>m 49	m 34<br>m 35<br>m 36<br>m 43<br>m 46<br>m 47<br>m 48	Trunk wash at m 42
3	–4 m<br>m 1	–	Trunk wash was not done	Necropsy at m 1: Lesional tissues (lung)
4	–	m 1<br>m 11<br>m 18<br>m 24<br>m 31<br>m 33	m 12<br>m 13<br>m 16<br>m 18<br>m 25<br>m 31<br>m 32	Trunk wash at m 24 (*Mycobaterium avium*)<br><br>Necropsy at m 33: Lesional tissues (trachea and mediastinal lymph nodes)

*Serologic testing by TB *Stat Pak* test (Chembio Diagnostic Systems, Inc, Medford, NY, USA), kindly provided by Dr Lyashchenko KP. m, month.

Technical Appendix Table 2. Clinical signs at and during hospitalization and gross and microscopic lesions as well as ZN-positive bacilli observed at and after necropsy*

Elephant ID	Clinical signs	Gross lesions	Microscopic lesions	ZN stain
1	Chronic weight loss, weakness, anorexia, dyspnea	Generalized lymphadenopathy, Purulent exudates and multifocal calcified pulmonary nodules. Hepatic congestion, together with renal multifocal micro abscesses.	Lung: severe chronic diffuse caseous necrosis, fibroplasias, infiltration of lymphocytes and macrophages.<br>Lymph nodes:Chronic diffuse caseous necrosis lymphadenitis.<br>Liver: severe centrilobular hemorrhage and necrosis multifocal caseous necrosis, diffuse lymphocyte and macrophage accumulation around blood vessels	Positive (100/HPF)
2<br>(still alive)	Good condition, serous nasal discharge	NA	NA	Positive (50/HPF)
3	Chronic weight loss, weakness, anorexia, depression	Multifocal calcified pulmonary nodules. Frothy, purulent exudates and ulcer in the upper trachea.	Lung: Chronic diffuse caseous necrosis, fibroplasias, Infiltration of lymphocytes and macrophages.<br>Lymph nodes: mild degree of lymphocytic depletion.	Positive (10/HPF)
4	Chronic weight loss, weakness, anorexia.	Pulmonary heamorrhage and edema, mediastinal lymph nodes enlargement, splenomegaly with multifocal micro abscesses.	Lung: pulmonary edema, infiltration of neutrophils.	Negative

*ZN, Ziehl-Neelsen staining for acid-fast bacilli in either of the samples of lung and mediastinal lymph nodes; HPF, high-power field; NA, not applicable.
